# Intermolecular β-Strand Networks Avoid Hub Residues and Favor Low Interconnectedness: A Potential Protection Mechanism against Chain Dissociation upon Mutation

**DOI:** 10.1371/journal.pone.0094745

**Published:** 2014-04-14

**Authors:** Giovanni Feverati, Mounia Achoch, Laurent Vuillon, Claire Lesieur

**Affiliations:** 1 Laboratoire d'Annecy-le Vieux de physique théorique (LAPTH UMR 5108), Université de Savoie, CNRS, Annecy le Vieux, France; 2 Laboratoire d'informatique systèmes, traitement de l'information et de la connaissance (LISTIC), Université de Savoie, Annecy le Vieux, France; 3 Laboratoire de mathématiques (LAMA UMR 5127), Université de Savoie, CNRS, Le Bourget du Lac, France; 4 Aging and imaging (AGIM FRE 3405), Université Joseph Fourier, CNRS, Grenoble, France; Swiss Institute of Bioinformatics, Switzerland

## Abstract

Altogether few protein oligomers undergo a conformational transition to a state that impairs their function and leads to diseases. But when it happens, the consequences are not harmless and the so-called conformational diseases pose serious public health problems. Notorious examples are the Alzheimer's disease and some cancers associated with a conformational change of the amyloid precursor protein (APP) and of the p53 tumor suppressor, respectively. The transition is linked with the propensity of β-strands to aggregate into amyloid fibers. Nevertheless, a huge number of protein oligomers associate chains via β-strand interactions (intermolecular β-strand interface) without ever evolving into fibers. We analyzed the layout of 1048 intermolecular β-strand interfaces looking for features that could provide the β-strands resistance to conformational transitions. The interfaces were reconstructed as networks with the residues as the nodes and the interactions between residues as the links. The networks followed an exponential decay degree distribution, implying an absence of hubs and nodes with few links. Such layout provides robustness to changes. Few links per nodes do not restrict the choices of amino acids capable of making an interface and maintain high sequence plasticity. Few links reduce the “bonding” cost of making an interface. Finally, few links moderate the vulnerability to amino acid mutation because it entails limited communication between the nodes. This confines the effects of a mutation to few residues instead of propagating them to many residues via hubs. We propose that intermolecular β-strand interfaces are organized in networks that tolerate amino acid mutation to avoid chain dissociation, the first step towards fiber formation. This is tested by looking at the intermolecular β-strand network of the p53 tetramer.

## Introduction

There exist proteins which function as oligomers by associating several copies of the same chains (homo-oligomers) or of different chains (hetero-oligomers). Chain association takes place through the formation of protein interfaces involving interactions between atoms of the amino acids of adjacent chains. Such intermolecular amino acid interactions are extensively studied by both experimental and computational approaches [1]–[5]. Alanine scanning mutagenesis have showed that only some of the amino acids of the interface account for the binding free energy [6]. Thus, there exists a subset of amino acids at interfaces, referred to as “hot spot” amino acids which are relevant for the chain association. This discovery has led to ample computational tool development aimed at identifying hot spots. The amino acids essential for interface formation are now known colloquially as hot spots, without necessarily implying alanine scanning validations.

Among proteins, some have the fold plasticity to undergo a transition from one oligomeric state to another. Of particular interest are the cases where the new oligomeric state impairs the protein function and leads to pathologies called protein misfolding diseases or conformational diseases. This transition is responsible for severe human diseases such as Alzheimer (Aβ-amyloid), Parkinson (synuclein) and cerebral amyloid angiopathy (cystatin C-amyloidosis). It is important to emphasize that the phenomenon is not restricted to neurodegenerative diseases but extends to cancer (p53), type II diabetes (IAPP, amylin), cardiovascular (transthyretin, serpin) and inflammatory diseases (Serpin) (reviewed in [7]–[11]). Note that in the previous sentence, for each of the diseases the protein undergoing the transition is indicated in brackets. A priori, these diseases are unrelated and the protein culprits do not share biological function, primary, secondary, tertiary or quaternary structures (initial or final). So the occurrence of the transition ought to be related to a local fold plasticity that allows transitions between different oligomeric states. It could be secondary structure plasticity as observed for the DIII loop of pore-forming toxins which becomes a β-hairpin and promotes the toxin's oligomerization or tertiary structure plasticity like the movement of the so-called “hinge loop” which leads to the formation of dimer or higher oligomeric states via a domain swapping mechanism [12]–[15].

The involvement of a local fold in the transition is in good agreement with the presence of a common structural motif in the pathological form of the culprit proteins. The pathological form, whether a fiber or an oligomer, involves interactions between two β-strands, each provided by a different chain (intermolecular β-strands). These intermolecular β-strands share several structural properties. They are recognized by the same antibody A11 [16]. Their formation depends on interactions between atoms of the backbone, result which has led to the proposal that aggregation is a generic property of the polypeptide chain [17], [18]. They adopt a cross β structure which can be predicted from sequences by the PIRA (Parallel ‘In Register’ Arrangement) model, a network made of single pairs of residues [19]–[24]. Different predictors of the aggregation-prone sequences involved in the fiber formation are now available [25]–[30].

Nevertheless, intermolecular β-strands are common in protein oligomers that are not known to undergo a transition to pathological assemblies. This suggests that there is a protection mechanism that prevents some intermolecular β-strands from undergoing the transition. We are interested in identifying the features pertaining to the vulnerability of intermolecular β-strands to undergo a transition to pathological assemblies. The intermolecular β-strand interactions that occur in conformational diseases are often referred to as “aberrant” interactions because they lead to a loss of protein function and finally to the disease while the intermolecular β-strand interactions that occur in “healthy” protein oligomers are referred to as “functional” interactions.

Previous studies mainly in dimers have shown that the frequencies of individual amino acids in intermolecular β-strands and in intramolecular β-strands are not different [31]. Yet we have reported that intermolecular β-strands of oligomers of quaternary structures above dimer, have a scattered charge distribution in contrast to intramolecular β-strands and “aberrant” β-strands which have charges confined to their C- and N-terminal extremities [26], [32], [33]. Edge β-strands have charges centrally located which prevent their aggregation, explanation that holds for intermolecular β-strands as well [34]. In our study, the individual hot spots did not have any features that could account for a transition from “functional” to “aberrant” β-strand interactions. Because of the small size of the dataset (40 intermolecular β-strands), it was not possible to investigate the properties of the hot spot pairs or of the layout of the interactions between hot spots.

We have now built a larger dataset of 1048 intermolecular β-strands enabling us to explore such properties. The results show that the hot spots are not matched randomly but according to chemical and geometrical properties of the side chains of the amino acids. The role of the geometry is novel and might open new venues to apprehend how intermolecular β-strands are formed. The main result is that the interactions between hot spots are organized to resist to the effects of amino acid mutation, possibly avoiding in this way chain dissociation upon mutation, first step to fiber formation.

## Results

The goal is to describe features of the hot spots involved in intermolecular β-strands and to consider how they may participate in a transition from “functional” to “aberrant” interactions. The intermolecular β-strands are represented as networks of hot spots in interaction with hot spots as nodes and interactions as links. Vocabulary related to graph and network theories are provided in methods.

### The tool Gemini

**Figure 1:**
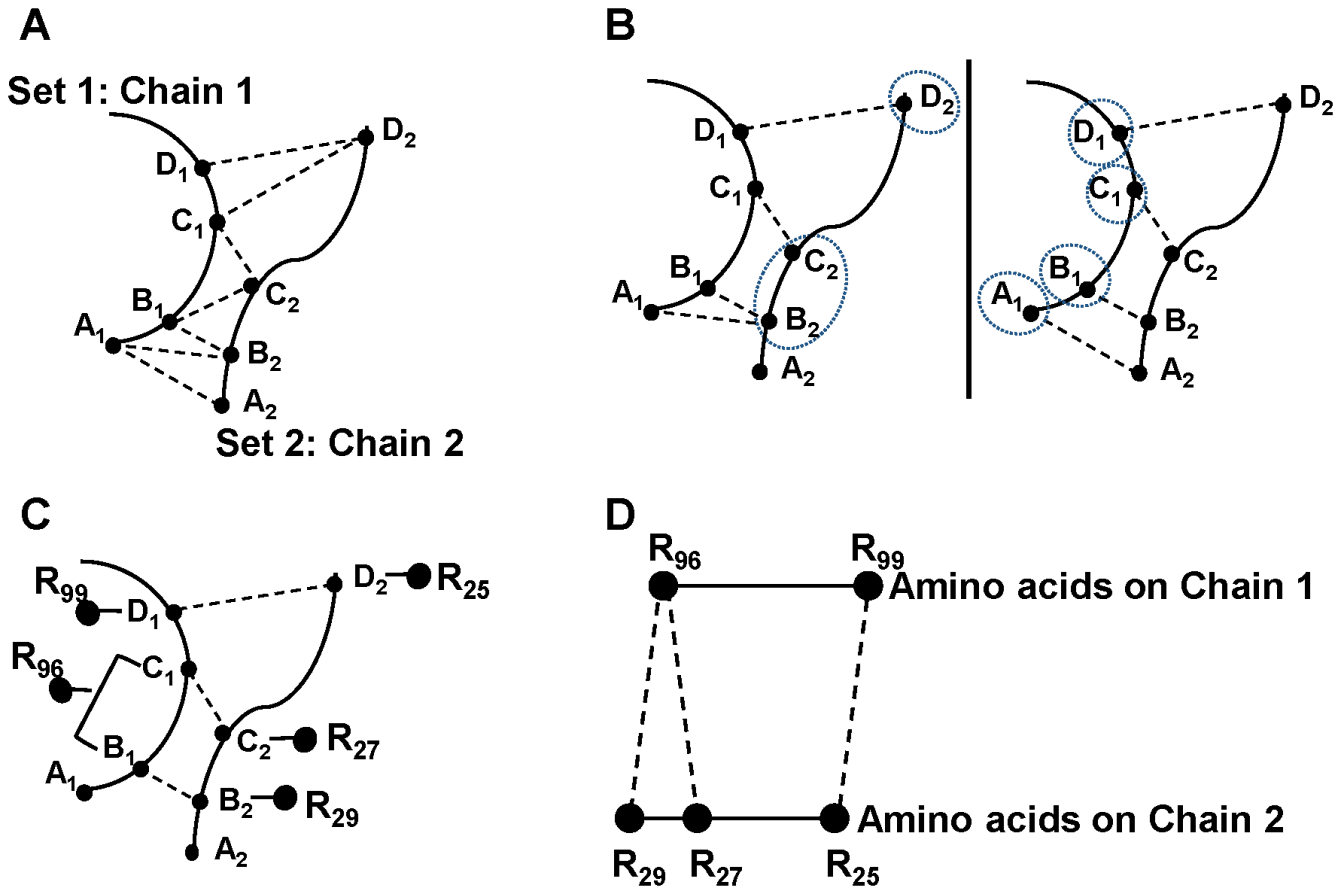
Illustration of the Gemini procedure on a trivial example. A. Interatomic distances between chain 1 and chain 2. On each chain, atoms are indicated by small filled circles labeled with letters. For clarity, only a few of the interatomic distances are indicated by dotted lines. B. Closest atoms. For every atom of S_1_, Gemini chooses the closest atoms on S_2_ (left picture) and for every atom of S_2_, Gemini chooses the closest atoms on S_1_ (right picture). The closest atoms are encircled. C. Mutually closest atoms. Gemini selects the atoms mutually the closest. The amino acids to which the mutually closest atoms belong are indicated by big filled circles. R stands for residue and the subscript is the position of the amino acid on the sequence. D. Gemini graph of amino acids in interaction. The distances between amino acids in contact are now arbitrary fixed to the same value because the information on the “real” interatomic distances is now lost. The pair of residues R99 and R25 is a single pair of amino acids (*k* = 1, that is one link connecting two residues). The residue R96 is a multiple contact amino acid because it is involved in two single pairs one with R29 and the other with R27, respectively.

The nodes and the links of the networks are identified by our tool Gemini. Gemini has been described previously, hence we only briefly recall how the networks are built [35], [36]. Each chain of a protein oligomer is considered as a set of points in the space whose positions are the Cartesian coordinates (x, y, z) of the atoms of the chain. The coordinates can be downloaded from the PDB. The atoms of the *chain 1* constitute the set 1 (S_1_) and the atoms of the *chain 2*, the set 2 (S_2_). Gemini calculates distances between every atom of S_1_ and every atom of S_2_ (interchain distances) but ignores the distances between atoms of a single set (intrachain distances) (Fig. 1A). Gemini chooses the closest atoms (Fig. 1B), and among them, retains only the pairs of mutually closest atoms (Fig. 1C). In other words, Gemini starts from an atom X_1_ of S_1_ and walks to its closest atom X_2_ on S_2_. It checks when coming back to S_1_ by the shortest distance that it retraces its step to X_1_. If not, the pair of atoms (X_1_, X_2_) is discarded, as for example for the pair (A_1_, B_2_) on figure 1C. The pairs of atoms that are mutually closest are considered to be interacting. At this stage the interchain interactions are symmetrical and the interface is referred to as around symmetrized [35]. In the last step, the pairs of atoms are replaced by their respective amino acids and a coarse-grained graph of amino acids in interaction is produced (Fig. 1D). Every amino acid has *k* interactions or *k* links where *k* equals to the number of atoms involved in a contact. There are single pairs of amino acids (*k* = 1, that is one link connecting two residues), multiple pairs of amino acids (*k* links connecting two residues) and multiple contact amino acids (an amino acid with *k* links to distinct amino acids).

Due to the choice of only mutually closest atoms, Gemini produces a graph of amino acids in interaction which is essentially a framework of interactions but not the set of all possible interactions. The amino acids selected by Gemini are detected as hot spots by available programs showing the robustness of defining an interface based only on geometry and its accuracy in picking up relevant amino acids [35]. It is important that Gemini does not need a cut-off distance to select atoms of the interface as classically done, for example to select preferentially backbone or side chain atoms. In this way Gemini avoids the variability of the selection inherent to the choice of a cut off [37]. Gemini naturally selects backbone and/or side chain atoms as part of the interface according to the geometry of the interface. Note that Gemini is applicable on any set of points in any metric space and can be used beyond the problem in question in the paper.

### The dataset

The PDBs of 755 protein oligomers containing at least one intermolecular β-strand interface are extracted from the RCSB (Biological assembly) and in total 1048 intermolecular β-strand networks are constructed with Gemini. It is a non-redundant dataset of oligomers assembling three (trimer) to twelve subunits (dodecamers). The oligomers are selected only on the presence of intermolecular β-strands since we are looking for elements relevant to the formation of the interface but not to the formation of the whole chain. To fit that condition and alleviate the pressure of evolution due to fold or function similarities, we need a dataset with high diversities in terms of the features of the whole chains. The 755 protein oligomers classify into 234 SCOP families, 30 distinct functions, are produced by organisms from the three domains of life and have on average a full chain length of 206±140 amino acids (average ± standard deviation) [38]–[40].

**Figure 2:**
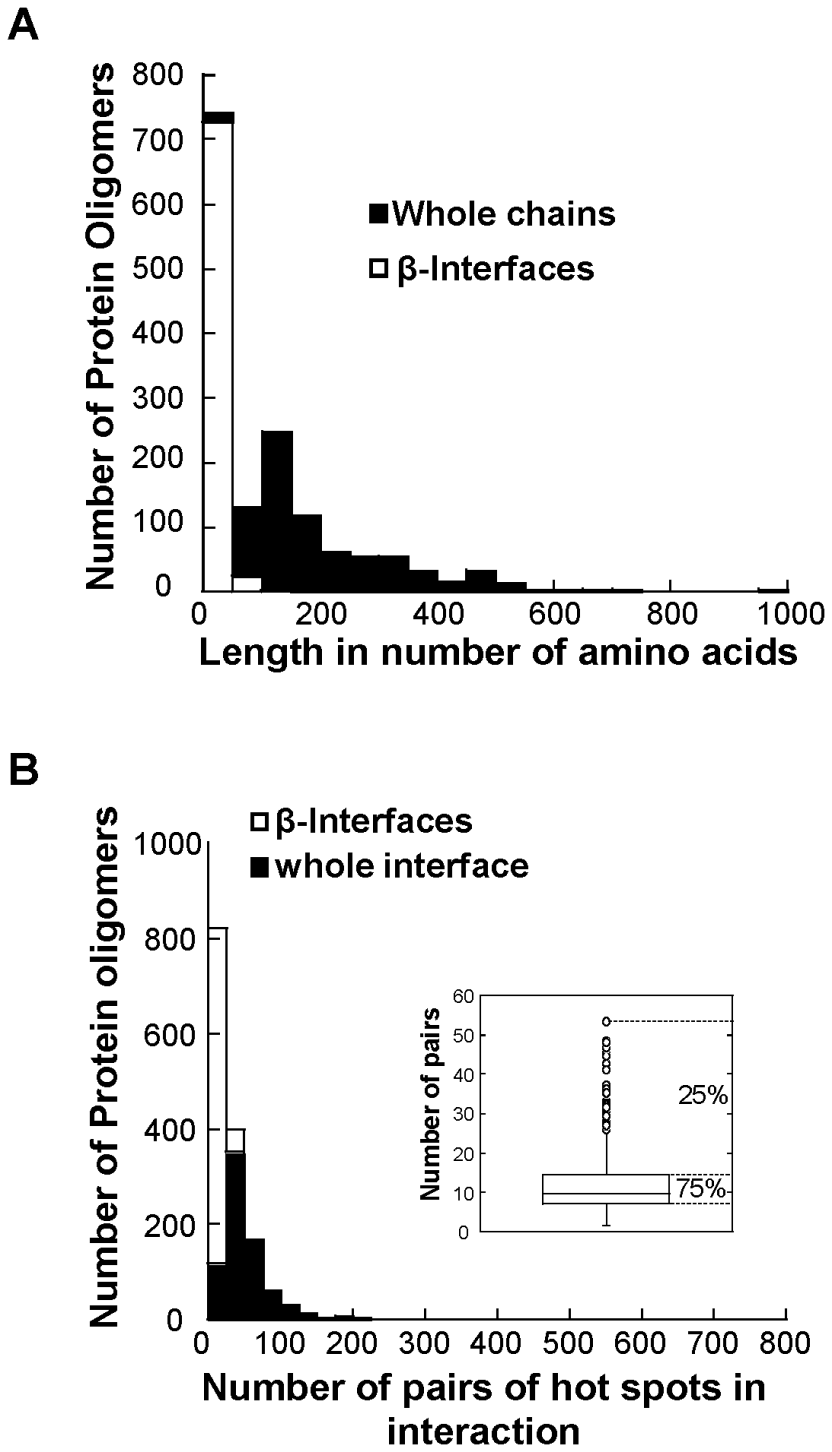
General features of the dataset. A. Histogram of the lengths (number of amino acids) of the whole chains (black bar) and of the intermolecular β-strands (white bar). B. Histogram of the number of hot spot pairs in the intermolecular β-strands (white bar) and in the whole interface (black bar). The inset is a box of the number of amino acid pairs in the intermolecular β-strands (quartile distribution). The values within the box (interquartile) represent 75% of the dataset. The points above the third quartile Q_3_ (outside of the box) are β-interfaces whose number of amino acid pairs deviates significantly from the rest of the dataset.

Now, on the contrary, we need a narrow diversity in terms of the features of the intermolecular β-strands to give evidences of a common construction mechanism. The average length of the intermolecular β-strands is 18±13 amino acids, length calculated as the sum of the amino acids of the two β-strands. The distribution of the whole chain lengths is broader than that of the β-interface lengths (Fig. 2A). The intermolecular β-strands have on average 13±8 hot spots, 75% have less than 16 hot spots and 25% have between 30 and 77 hot spots. Likewise, there are on average 12±8 hot spot pairs per interface, 75% of the interfaces have less than 15 hot spot pairs while 25% have between 25 to 50 (Fig. 2B, inset). The number of hot spot pairs in the intermolecular β-strands is compared to the total number of hot spots pairs over the whole interfaces to assess the diversity of intermolecular β-strands in terms of the number of interactions necessary to build them. The distribution of the number of pairs in intermolecular β-strands is narrower than in the whole interface (Fig. 2B). Globally, 75% of the dataset have intermolecular β-strands sharing features. Moreover, there is no correlation between the length of the whole chain and the length of the intermolecular β-strands (not shown, R = 0.03) supporting the idea that the two objects have independent features.

The dataset contains 568 anti-parallel β-sheets, 132 parallel β-sheets and 348 other β-strand arrangements (close packed β-strands) and 60% of the cases have β-strands with distinct sequences. One can already anticipate that the intermolecular β-strands of the dataset cannot be predicted based on a network of pairs of residues following a Parallel In Registered Arrangement (PIRA) because only 12% are parallel β-sheets and most β-strands have non identical sequences. The global features already highlight a network arrangement different from aggregation prone sequences [25].

### Analysis of the properties of the residues in interaction in intermolecular β-strands

Gemini labels backbone and side chain atoms of the amino acids such that it produces two sub-graphs: one involving pure backbone interatomic interactions (BB networks) and the other involving interactions with at least one atom of the side chain (SC networks). We have shown that this distinction is necessary to exhibit features of intermolecular β-strands [32]. This is certainly related to the involvement of the backbone interactions in the hydrogen bond network of the β-sheets while in α-helices such backbone interactions are involved intramolecularly and are not interfering with intermolecular interactions. This is in good agreement with previous reports that side chain and backbone interactions are involved in hydrophobic and hydrogen bonding, respectively [1], [41], [42].

First, the properties of the individual hot spots are analyzed. Totals of 704623, 10692 and 5950 amino acids are observed in the whole chains, the SC and the BB hot spots, respectively. These figures give evidences of the reliability of the statistics which improves with the size of the sample. The amino acid frequencies are indicated in table 1 and used to measure the average chemical property, the global (GP) and local propensity (LP) of the amino acids (Tables 2 and 3, respectively). As observed previously the SC and BB hot spots have average chemical properties similar to the amino acids of the whole chains, global propensity and local amino acid distribution coherent with sequences made of β-strands as well as a scattered charge distribution [32]. Namely high β-sheet propensity residues (F, W, Y) are significantly more frequent while low β-sheet propensity (G and A) are significantly less [43]–[45]. The β-strand extremities are enriched in β-breaker amino acids (P and G) while high β-sheet propensity residues are enriched centrally (V, L) [46], [47]. The charged residues R, K and E are enriched at the β-strand extremities whereas H and D residues are more frequent centrally when the local preferences of the SC charged residues is considered (Table 4).

**Table 1:** Whole chain amino acid and individual hot spot frequencies.

Amino acid	Whole chain	SC	BB
A	0.086	0.037	0.057
C	0.012	0.009	0.016
D	0.057	0.048	0.034
E	0.071	0.066	0.052
F	0.039	0.057	0.053
G	0.078	0.032	0.081
H	0.023	0.033	0.021
I	0.064	0.074	0.096
K	0.058	0.053	0.050
L	0.088	0.080	0.081
M	0.018	0.024	0.024
N	0.038	0.043	0.032
P	0.045	0.040	0.012
Q	0.033	0.040	0.037
R	0.051	0.062	0.050
S	0.056	0.062	0.061
T	0.056	0.075	0.070
V	0.083	0.090	0.109
W	0.011	0.019	0.013
Y	0.032	0.056	0.050

**Table 2:** Chemical properties of the intermolecular β-strands and of the whole chains (%).

Cases	Hydrophobic	Charged	Polar
**Whole chain residues**	49±5	26±5	25±6
**BB hot spots (all)**	58±27	19±20	23±23
**BB hot spots (anti-parallel)**	59±26	19±19	21±21
**BB hot spots (parallel)**	58±17	17±15	25±18
**SC hot spots (all)**	47±20	26±19	26±17
**SC hot spots (anti-parallel)**	47±21	26±19	26±18
**SC hot spots (parallel)**	46±17	25±16	29±14

**Table 3:** Global propensities and local preferences of the SC and BB hot spots.

	SC hot spots				BB hot spots			
Amino acid	Global propensity	Outer frequency (*f_O_*)	Central frequency (*f_C_*)	*f_O_*–*f_C_*	Global propensity	Outer frequency (*f_O_*)	Central frequency (*f_C_*)	*f_O_*–*f_C_*
**A**	0.4	0.04	0.04	−0.003	0.7	0.06	0.05	0.009
**C**	0.8	0.01	0.01	−0.006	1.3	0.01	0.02	−0.012
**D**	0.8	0.05	0.05	−0.003	0.6	0.04	0.03	0.005
**E**	0.9	0.07	0.06	0.012	0.7	0.05	0.05	0.002
**F**	1.5	0.06	0.06	−0.004	1.4	0.05	0.06	0.003
**G**	0.4	0.03	0.03	0.002	1.0	0.09	0.07	0.02
**H**	1.4	0.03	0.03	−0.003	0.9	0.02	0.02	−0.004
**I**	1.1	0.07	0.08	−0.007	1.5	0.09	0.1	−0.004
**K**	0.9	0.06	0.05	0.014	0.9	0.06	0.05	0.011
**L**	0.9	0.08	0.08	0.001	0.9	0.07	0.09	−0.018
**M**	1.3	0.02	0.02	−0.003	1.3	0.02	0.03	−0.003
**N**	1.1	0.04	0.04	−0.002	0.8	0.04	0.03	0.013
**P**	0.9	0.05	0.03	0.017	0.3	0.02	0.01	0.009
**Q**	1.2	0.04	0.04	−0.002	1.1	0.04	0.04	0.005
**R**	1.2	0.08	0.05	0.025	1.0	0.05	0.05	0.006
**S**	1.1	0.06	0.07	−0.008	1.1	0.06	0.07	−0.012
**T**	1.4	0.07	0.08	−0.011	1.3	0.07	0.07	−0.004
**V**	1.1	0.08	0.1	−0.023	1.3	0.10	0.12	−0.019
**W**	1.7	0.02	0.02	−0.002	1.1	0.013	0.013	0.000
**Y**	1.8	0.06	0.05	0.005	1.6	0.05	0.05	−0,003
**Average**	1.1	0.05	0.05	0.000	1.0	0.05	0.05	0.000
**S.D.**	0.4	0.02	0.02	0.011	0.3	0.03	0.03	0.01

**Table 4:** Local preferences of the charged amino acids in the SC hot spots.

Charged	Outer frequency (*f_O_*)	Central frequency (*f_C_*)	*f_O_*–*f_C_*
**D**	0.16	0.20	−0.042
**E**	0.25	0.25	0.004
**H**	0.11	0.14	−0.033
**K**	0.21	0.20	0.018
**R**	0.27	0.21	0.053
**Average**			0.000
**S.D.**			0.039

**Figure 3:**
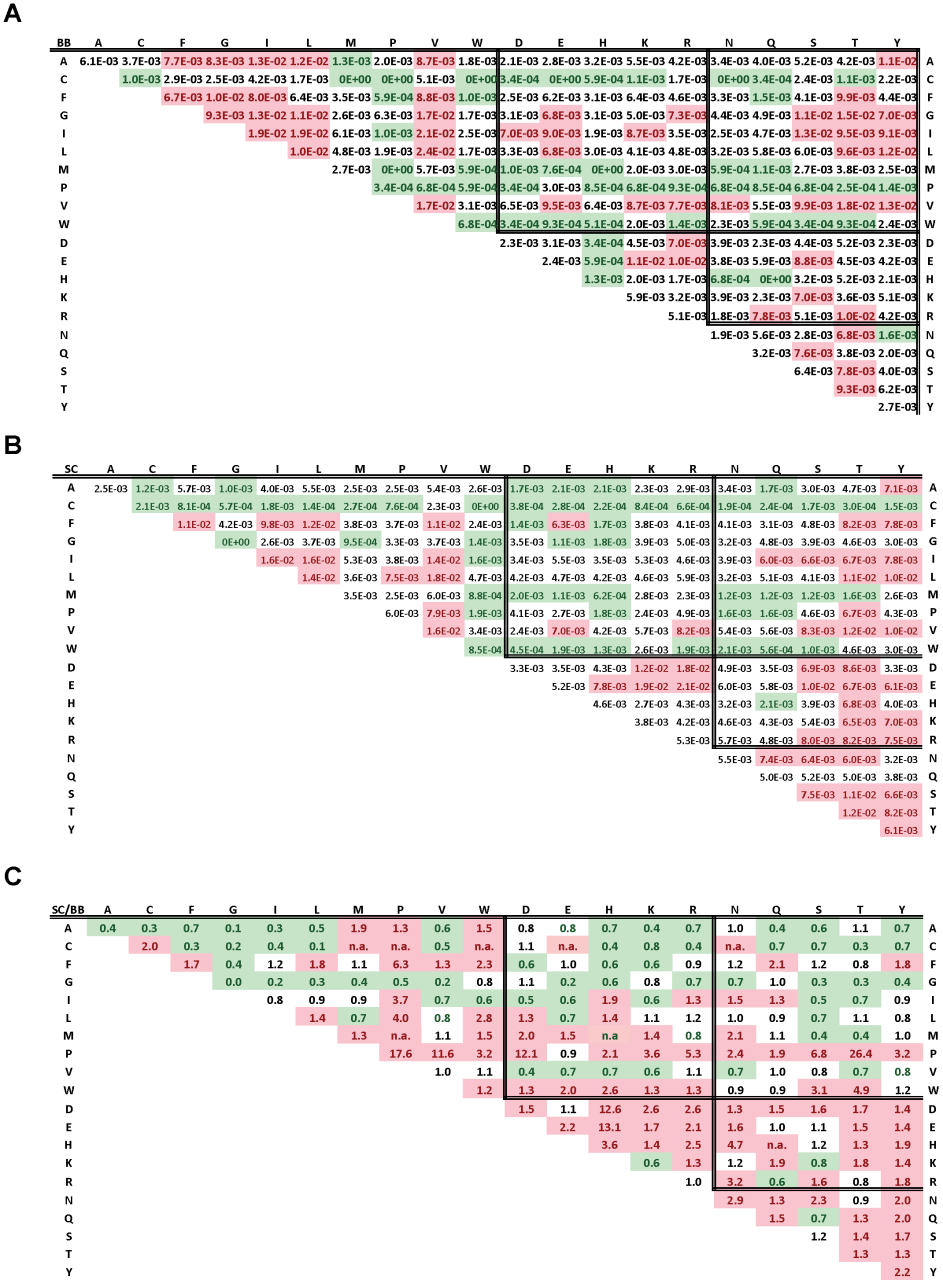
Tables of the *f_ab_* pair frequencies. A. Observed BB pair frequencies. B. Observed SC pair frequencies. The frequency *fab* is for pairs of hot spots *ab* read on the lines *a* and the columns *b*. The preferred (>Q_3_) and avoided (<Q_1_) pairs are indicated by red and green color, respectively. The pairs with a frequency between Q_1_ and Q_3_ are not colored. The residues are ordered alphabetically within hydrophobic, charged and polar groups. C. SC and BB pair distinction. The ratios of the frequency of a pair *ab* in the SC sub-networks to its frequency in the BB sub-networks are indicated. The pairs more frequent in the SC sub-networks are indicated in red (ratio >1.2) and the pairs more frequent in the BB sub-networks are indicated in green (ratio <0.8). For ratio ranging from 0.8 to 1.2, the pairs are not colored. The abbreviation n.a. stands for “not applicable” which is division per zero, those pairs are more represented in the SC sub-networks.

Second, the properties of the pair of hot spots in interaction are analyzed. Because most of the intermolecular β-strands are not made of β-strands with an identical sequence, the occurrences *n_ab_* and *n_ba_* are initially counted but a χ^2^ test calculated over the occurrences *n_ab_* and *n_ba_* shows that the differences are insignificant and so *n_ab_* and *n_ba_* occurrences are summed (Tables 5 and 6). The test ignored the values for the pair of identical residues for which *a* equals *b*. There are 10551 SC pairs and 5894 BB pairs, again highlighting the reliability of the statistics. The frequencies of the hot spot pairs *f_ab_* are calculated with equation (1) and shown in the tables constituting the figure 3.

**Table 5:** Observed BB Pair occurrences.

BB	A	C	D	E	F	G	H	I	K	L	M	N	P	Q	R	S	T	V	W	Y	∑ 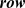	∑ 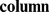	Average	Chi^2^
**A**	36	11	5.5	7	23	24	8.5	39.5	16.0	34.0	4.0	10	5.5	11.5	13	15	12.5	25.5	5.5	32	303.0	309.7	306.3	0.8
**C**	11	6	1	0	8	7.5	2	12	2.5	5	0	0	0	1	5	6	3.5	16.5	0	6	8.7	89.5	88.3	0.9
**D**	7	1	13.5	10	8	8.5	1	23	15	10	3.5	11	1	6.5	20	13.5	15	21	1	8	184	168	176	0.4
**E**	9.5	0	8.5	14	19.5	20	1.5	27.5	31.5	19.5	2	12	8.5	17	30.5	26	14.5	28.5	3	13	292.5	285.8	289.2	0.8
**F**	22.5	9	7	17	39.5	28.5	8.5	23.5	19	19	9.5	10	1.5	4.5	14	11	29.5	26	3	13.5	276.5	285.7	281.1	0.7
**G**	24.7	7.5	10	20.3	32.7	54.7	9	37.5	14.5	32.8	8	13.5	18.5	15.2	21	34	48.8	51.8	5	22	426.8	405.8	416.3	0.5
**H**	10.5	1.5	1	2	9.5	9	7.5	5.5	6	8.5	0	2	2	0	5	10	14.5	19.5	2	6	114.5	111	112.8	0.8
**I**	39.5	13	18.5	25.5	23.5	41	5.5	111.5	23.5	52.5	18	6.5	3	14.5	8.5	37.5	28.5	60	7	27.5	453.5	474.5	464	0.5
**K**	16.5	4	11.5	34	19	15	5.5	27.5	35	12	6	12	2	7	9.5	18.5	11	27.5	6	15	259.5	252.5	256	0.8
**L**	35	5	9.5	20.5	19	32	9	57.5	12	61	13.5	9	5	16	13	18	28.5	70.5	5.5	36.5	415	412.7	413.8	0.9
**M**	3.5	0	2.5	2.5	11	7.5	0	18	6	15	16	2	0	3.5	9	7.5	11.5	16.5	2	8	126	122.5	124.3	0.8
**N**	10	0	12	10.5	9.5	12.5	2	8.5	11	10	1.5	11	2	16.5	5	8.5	20	26.5	7	5	178	172	175	0.7
**P**	6	0	1	9	2	18.5	3	3	2	6	0	2	2	2.5	3	2	1	2	2	4	69	64	66.5	0.7
**Q**	12	1	7	17.5	4.5	14	0	13	6.5	18	3	16.5	2.5	19	23.5	21	12	17	2	7	198	193.7	195.8	0.8
**R**	11.5	5	21	30	13	22	5	12	9.5	15	8.5	5.5	2.5	22.5	30	15	29	23.5	5	12.5	268	263	265.5	0.8
**S**	15.5	8	12.5	26	13	33.3	9	40.5	22.5	17.5	8.5	8	2	23.5	15	37.5	22.7	31.5	1	12.5	322.5	305.8	314.2	0.5
**T**	12.5	3	15.5	12	29	37	16	27.5	10	28	11	20	.5	10.5	30.5	23.5	55	54.5	3	18	362	374	368	0.7
**V**	26	13.5	17.5	27.5	26	51	18	64.5	24	70	17	21	2	15.5	22	27	50.5	99.5	8.5	39.5	541	566.7	553.8	0.4
**W**	5	0	1	2.5	3	5	1	8	6	4.5	1.5	6.5	1.5	1.5	3.5	1	2.5	9.5	4	7	70.5	75.5	73	0.7
**Y**	31.5	7	5.5	12	12.5	19.5	6.5	26	15	35.3	7	4.5	4	4.5	12	10.8	18.5	38.8	7	16	278	293	285.5	0.5

The values in a given row are the occurrences of the residue *a* in contact with the residues *b_i_*, cited on columns. Thus, there are twenty rows *a_i_* and twenty column *b_i_,* - *i*- covering the 20 different amino acids. Due to the counting procedure the table is read row-wise (material and methods).

**Table 6:** Observed SC Pair occurrences.

SC	A	C	D	E	F	G	H	I	K	L	M	N	P	Q	R	S	T	V	W	Y	∑ 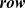	∑ 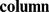	Average	Chi^2^
**A**	26.3	7.3	9.2	11.7	35	5	12.5	22.8	13	35	15	18	15	9.8	17	15.8	26.5	32.2	14.5	44	359.3	288.6	324	0
**C**	5.3	22	2	2	5	3	1.5	11	4.5	1	1.3	1	4.5	1	4	8.5	1.3	12	0	9	78	72.3	75.2	0.6
**D**	8.8	2	35.3	19.3	8.3	17	23	18.7	64.5	21.8	10.5	25.8	20.7	20	101.5	33.8	44	11.8	2.8	18.7	473.2	460.6	466.9	0.7
**E**	11	1	17.9	54.8	34.4	4.8	42.7	28.3	102.3	24	5.5	28.4	14.7	29.7	120	49.4	33.6	35.3	11.3	37	631.3	628.5	629.9	0.9
**F**	24.6	3.5	6.8	32.5	120	18.3	9.6	48.1	20.2	55.7	18.9	21.7	17.3	16.1	21.7	21.7	41.8	55.4	12.7	43.2	489.5	553.2	521.4	0
**G**	5.5	3	20	7	26.5	0	11	15.3	25	22.5	5.5	17.8	20	27.5	35.3	20.8	25.5	21	8.7	20	338	255.2	296.6	0
**H**	10	0.8	22.2	39.2	8.8	8.3	48.5	16.7	12.5	20.7	3	15.7	8.7	9.8	24.2	19.7	35.3	21.3	7.6	21.8	306.2	334.1	320.2	0.3
**I**	19.8	8.5	17.2	29.9	55.5	12.4	19.8	169.1	28	83.2	30.5	21.3	21.7	35.3	24.9	33	33.5	76.2	8.3	43.4	602.4	593	597.7	0.8
**K**	10.8	4.3	60	99.8	20.1	16.7	16.5	28.4	40.3	23.9	14.5	22.6	115	25	24	24.8	37.8	28.6	13.8	38.5	525.2	530	527.6	0.9
**L**	23	.5	23	25.4	68.3	16.1	24	89.5	25	149.9	18.8	17.5	41.2	26.3	35.7	18.3	56	94.6	29.7	57.3	690.1	662.4	676.2	0.5
**M**	11.1	1.5	11.1	6.4	20.9	4.5	3.5	25.2	14.9	18.8	36.8	6.1	12	7	11.5	5	8.9	27.3	4.3	15.2	215.2	231.7	223.5	0.4
**N**	17.7	1	25.7	34.7	21.7	16.1	18	20.2	25.5	16.1	6.8	57.6	8.5	38.7	32	32.8	31.5	26.7	12	15.8	401.4	398	399.7	0.9
**P**	11.2	3.5	22.7	13.5	22.2	14.3	10	18.5	10.8	38.3	14.3	8.8	63	8.5	28	22	33.5	41.7	11.8	25.8	359.5	364.6	362	0.8
**Q**	8.5	1.5	17.2	31.8	17	22.7	12	28.2	20.2	27.1	5.7	39.3	8.2	52.3	26.5	25.3	24.5	25	3.3	20.2	364.2	392.6	378.4	0.3
**R**	13.3	3	88.7	106.4	21.8	17.5	21.3	23.4	20.3	26.7	12.5	28	24.2	24.1	56.3	38.3	40.3	39.3	10	39.7	598.7	694.8	646.7	0
**S**	15.7	9.5	39	57.3	28.5	20.5	22	36.8	32.5	24.5	7.8	34.3	27	29.5	46	79.5	58.5	45.3	6.5	39.3	580.5	500.3	540.4	0
**T**	22.7	1.8	46.5	36.7	44.8	22.6	36.7	37.5	30.8	56.9	7.9	32.2	37.3	27.8	46.5	54.3	127.1	65.5	22.8	46	677.4	661.2	669.3	0.7
**V**	25.3	12.5	13.8	38.8	62.7	17.8	23.2	76.6	31.2	97.9	36	30.8	41.3	34	47.3	42.4	63.2	170.8	21.3	56.3	772.3	723.5	747.9	0.2
**W**	13.4	0	2	8.7	12.5	6.3	6.2	8.6	13.2	20.1	5	10.5	8.1	2.5	9.7	4.5	25.4	14.6	9	15.3	186.5	217.5	202	0.1
**Y**	30.8	7	15.8	27.2	39.2	11.5	20.8	39.4	35.6	48.2	12.1	18.3	19.3	20	39.1	29.8	40.1	49.8	16	64.1	519.9	606.4	563.2	0

The values in a given row are the occurrences of the residue *a* in contact with the residues *b_i_*, cited on columns. Thus, there are twenty rows *a_i_* and twenty column *b_i_,* - *i*- covering the 20 different amino acids. Due to the counting procedure the table is read row-wise (material and methods).



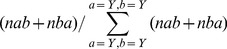
(1)The ratio (*f_ab_*/*f_a_.f_b_*) is measured to compare observed values *f_ab_* with expected values (*f_a_.f_b_*) (Tables 7 and 8). If the frequency *f_a_* is independent of the frequency *f_b_* the ratio is equal to one. Overall the hot spots are not matched randomly since 70% and 66% of the BB and SC pairs, respectively, have a ratio that deviates from one. It is therefore necessary to measure the pair frequencies because they cannot be simply derived from the frequencies of individual hot spots.

**Table 7:** Ratio of f_ab_/(f_a_.f_b_) for the BB hot spot pairs.

f_ab_/f_a_.f_b_	A	C	F	G	I	L	M	P	V	W	D	E	H	K	R	N	Q	S	T	Y
**A**	2	4	3	2	2	3	1	3	1	2	1	1	3	2	1	2	2	1	1	4
**C**		4	3	2	3	1	0	0	3	0	1	0	2	1	2	0	1	3	1	3
**F**			2	2	2	1	3	1	2	2	1	2	3	2	2	2	1	1	3	2
**G**				1	2	2	1	6	2	2	1	2	2	1	2	2	2	2	3	2
**I**					2	2	3	1	2	2	2	2	1	2	1	1	1	2	1	2
**L**						2	2	2	3	2	1	2	2	1	1	1	2	1	2	3
**M**							5	0	2	2	1	1	0	2	2	1	1	2	2	2
**P**								2	1	4	1	5	3	1	2	2	2	1	0	2
**V**									1	2	2	2	3	2	1	2	1	2	2	2
**W**										4	1	1	2	3	2	6	1	0	1	4
**D**											2	2	0	3	4	4	2	2	2	1
**E**												1	1	4	4	2	3	3	1	2
**H**													3	2	2	1	0	3	4	2
**K**														2	1	2	1	2	1	2
**R**															2	1	4	2	3	2
**N**																2	5	1	3	1
**Q**																	2	3	1	1
**S**																		2	2	1
**T**																			2	2
**Y**																				1

**Table 8:** Ratio of f_ab_/(f_a_.f_b_) for the SC hot spot pairs.

f_ab_/f_a_.f_b_	A	C	F	G	I	L	M	P	V	W	D	E	H	K	R	N	Q	S	T	Y
**A**	2	3	3	1	1	2	3	2	2	4	1	1	2	1	1	2	1	1	2	3
**C**		24	1	2	3	0	1	2	3	0	1	0	1	2	1	0	1	3	0	3
**F**			3	2	2	3	3	2	2	2	1	2	1	1	1	2	1	1	2	2
**G**				0	1	1	1	3	1	2	2	1	2	2	3	2	4	2	2	2
**I**					3	3	3	1	2	1	1	1	1	1	1	1	2	1	1	2
**L**						2	2	2	3	3	1	1	2	1	1	1	2	1	2	2
**M**							6	3	3	2	2	1	1	2	2	1	1	1	1	2
**P**								4	2	3	2	1	1	1	2	1	1	2	2	2
**V**									2	2	1	1	1	1	1	1	2	1	2	2
**W**										2	1	2	2	3	2	3	1	1	3	3
**D**											1	1	3	5	6	2	2	2	2	1
**E**												1	4	5	5	2	2	2	1	2
**H**													4	2	2	2	2	2	3	2
**K**														1	1	2	2	2	2	2
**R**															1	2	2	2	2	2
**N**																3	4	2	2	1
**Q**																	3	2	2	2
**S**																		2	2	2
**T**																			2	2
**Y**																				2

**Figure 4:**
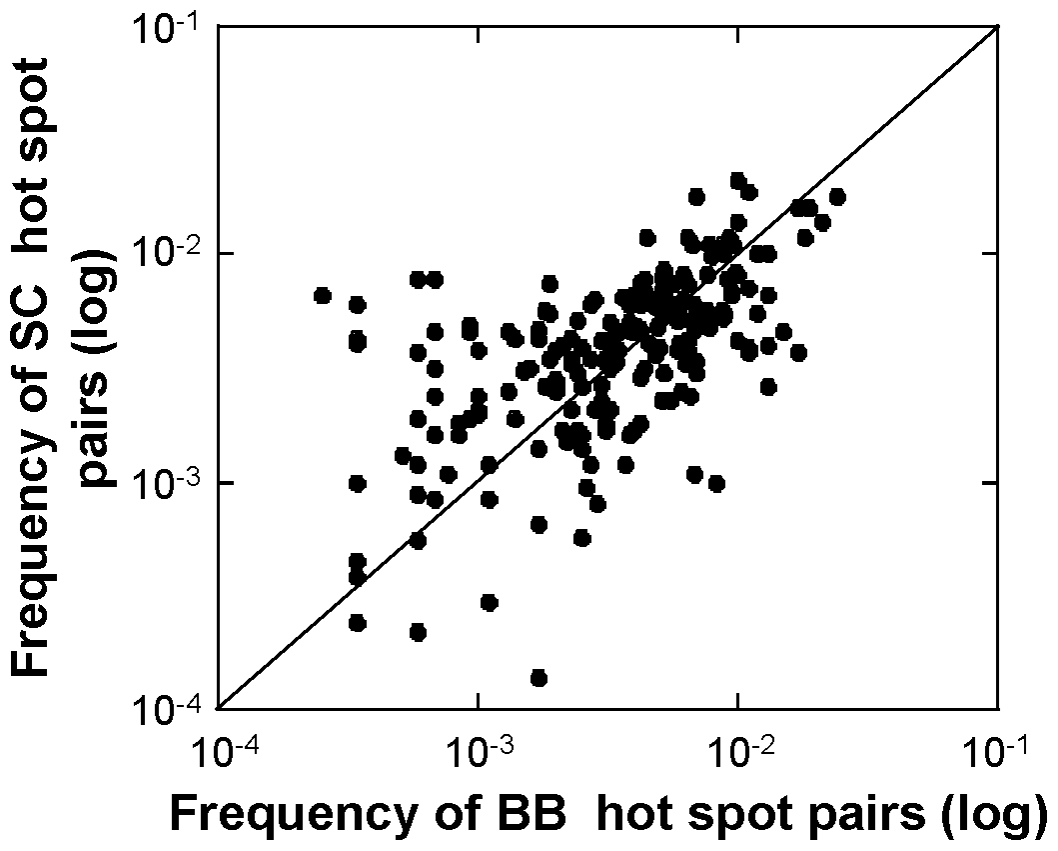
Comparison of the 210 frequencies of the BB and SC hot spot pairs. The frequencies of the SC hot spots pairs are plotted against those of the BB hot spots pairs, both in log scale. Pairs with identical BB and SC frequencies are on the diagonal. Pairs more frequent in SC are found above the diagonal whereas pairs more frequent in BB are found below the diagonal.

To evaluate if the distinction between SC and BB hot spots is also relevant at the level of the pairs, the frequencies of the SC pairs are plotted against the frequencies of the BB pairs (Fig. 4). On the diagonal, there are 50 pairs out of a total of 210, thus indicating that 76% of the BB and SC pairs have different frequencies. It is therefore important to investigate them separately. Subsequent analyses are performed using quartiles to take into account the observation that 75% of the intermolecular β-strands share similar global interface features while 25% are more heterogeneous The amino acids with the highest 25% pair frequencies (> quartile Q_3_) are considered as preferred contacts (Fig. 3, red) whereas those with the lowest 25% pair frequencies (<quartile Q_1_) are considered as avoided contacts (Fig. 3, green). The neutral contacts have the frequencies between Q_1_ and Q_3_ (Fig. 3, white). The Q_3_ and Q_1_ of the SC hot spot pairs are 6.0×10^−2^ and 2.2×10^−3^, respectively. The Q_3_ and Q_1_ of the BB hot spot pairs are 6.7×10^−2^ and 1.7×10^−2^, respectively. In both networks, on average every amino acid pairs with 5 other types of amino acids out of its twenty pairing possibilities. The most preferred contacts are measured as amino acids which pair with a frequency above Q_3_ with more than five other types of amino acids. For both networks, the most preferred contacts are I, L, V, S and T similarly to what was found for intermolecular β-strands in dimers [31]. On the other hand compared to the dimers F and Y residues are preferred in the SC networks while A and G are preferred in the BB networks, the residue E is preferred in both. Likewise, the most avoided contacts are measured as amino acids which pair with a frequency below Q_1_ with more than five other types of amino acids. For both networks, the most avoided contacts are with C, M, W and H residues, similarly to intermolecular β-strands in dimers. In addition contacts with A, G and Q are avoided in the SC networks while contacts with N and Q residues are avoided in the BB networks.

The features of the hot spots pairs are then analyzed considering the chemistry and the geometry of amino acids (Tables 9 and 10, respectively). Both SC and BB hot spot pairs have similar tendencies for contacts with hydrophobic residues but the contacts with polar and charged residues are twice more frequent in the SC pairs. Even more blatant differences are the contacts between two charged residues, or between two polar residues or else between one charged and one polar residue, at least ten times more frequent in the SC networks. Considering geometrical properties (length of the side chains) the contacts with long and medium residues are significantly more frequent in the SC pairs than in the BB ones which on the contrary favor contacts between short side chain residues.

**Table 9:** SC and BB hot spot pair chemical tendencies.

Pair property	Total	SC tendency	BB tendency	Neutral
**(Fhi, X)**	155	58	65	32
**(Ch, X)**	90	50	26	14
**(P, X)**	90	46	22	22
**(Fhi, Fhi)**	55	24	23	8
**(Fhi, Ch)**	50	19	23	8
**(Fhi, P)**	50	15	19	16
**(Ch, Ch)**	15	12	1	2
**(Ch, P)**	25	19	2	4
**(P, P)**	15	12	1	2

The number of pairs with a ratio SC pair frequency to BB pair frequency above 1.0±0.2 indicates the SC pair tendency. The number of pairs with a ratio below 0.8±0.2 indicates the BB pair tendency (table based on Fig. 2C). The second column, total, indicates the pair combinatory of the chemical pair property mentioned in the first column. Fhi, Ch and P stand for hydrophobic, charged and polar residues. X stands for fhi, ch and P.

**Table 10:** SC and BB hot spot geometrical pair tendencies.

Geometrical pair property	Total	SC preferred	BB preferred	Neutral
**(L, X)**	**74**	**43**	**10**	**21**
**(M, X)**	**144**	**72**	**41**	**31**
**(S, X)**	**119**	**41**	**49**	**29**
**(L, L)**	**10**	**8**	**1**	**1**
**(L, M)**	**36**	**23**	**5**	**8**
**(L, S)**	**28**	**12**	**4**	**12**
**(M, M)**	**45**	**29**	**6**	**10**
**(M, S)**	**63**	**20**	**30**	**13**
**(S, S)**	**28**	**9**	**15**	**4**

Legend as in table 9. L, M and S stand for long, medium and short side chains. × stands for L, M and S.

**Figure 5:**
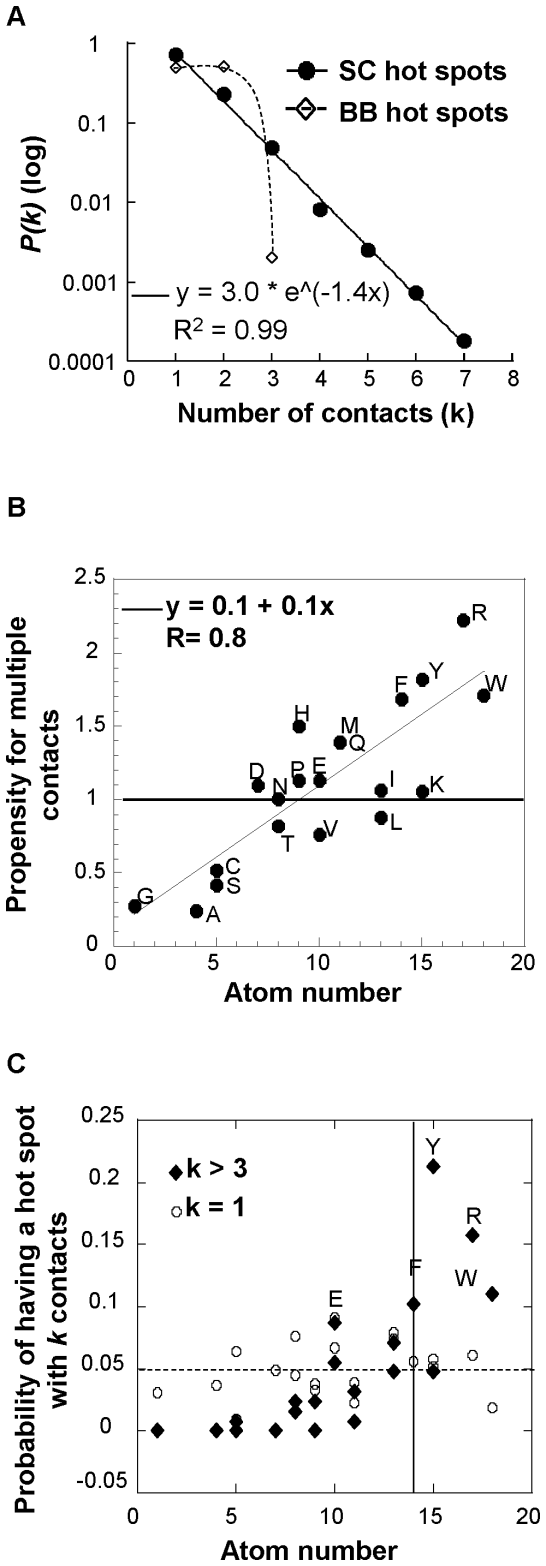
Number of contacts of the hot spots. A. The degree distributions of the BB and SC hot spots are plotted on a semi-log scale. The degree distribution *P(k)* of the SC hot spots decreases exponentially (R^2^ = 0.99). B. Linear correlation between the number of atoms of a SC hot spot and its tendency to have more than one contact. The ratio of the frequency of an amino acid in multiple contacts to its frequency in single contact is plotted against the number of its side chain atoms. C. Probability of a SC hot spot to have *k* contacts. The probabilities for a SC hot spot to have *k*>3 (♦) or *k* = 1 (○) are plotted against the number of atoms of its respective amino acid. The horizontal line indicates the probability at which every amino acid has the same probability to have *k* contacts (0.05 = 1/20). The vertical line indicates a number of atoms equals to 14.

Third, the number of contacts of the hot spots is counted to determine whether the hot spots have multiple contacts. The BB networks have as many single contact hot spots (2941) as two contact hot spots (2993) but very little three contact hot spots (12). The degree distribution *P(k)* is equal to the ratio of the number of hot spots with *k* contacts to the total number of hot spots. For the BB networks, *P(k)* has a bell-like shape with an average <*k*> contacts equals to 1.5 (Fig. 5A). On the other hand, *P(k)* for the SC networks falls on a straight line when plotted on a linear-log scale indicating an exponential decay, a variation from the power law distribution observed for real networks [48] (Fig. 5A, R ^2^ = 0.99). The average <*k*> contact of the SC hot spots is 1.4.

To determine a prototype intermolecular β-strand network, we use a binomial model with 9 amino acids per strand, 6 hot spots and the probability *p* = 0.16 of having a contact (see methods for definition of a binomial law). These values are based on the averages of 18 amino acids, 12 hot spots and 10 links per interface measured over the dataset. A fully connected graph of 9 amino acids per strand (all amino acids have at least one link with all others) would have 81 links (9 by 9) and so in total on the dataset 84888 links. Only 13628 links are measured, thus the probability *p* of making a contact (having a link) is equal to 0.16 (13628/84888). Assuming that the amino acids have a uniform distribution of links (i.e. all amino acids have the same probability of making a link), the binomial model calculates a prototype network with 21% of non-connected amino acids (not hot spots), 36% of amino acids with one contact and 43% of amino acids with more than one contact, 27% of amino acids would have two contacts and 12% would have three. The observed data indicate 49% of amino acids with one contact and 51% amino acids with more than one contact, 33% with two, 14% with three and 4% with more than 3 contacts. The observed data are measured on hot spots only and so do not take into account the non-connected amino acids. In the binomial model, the “hot spots”, namely the amino acids with a link are 79% (36% with one contact and 43% with more than one). The percentage of amino acids with *k* contacts over a network made only of hot spots can be estimated for the binomial model by multiplying the calculated values by a factor 100/79. That produces 46% of hot spots with one contact (36 * 100/79), 54% (27*100/79) of hot spots with more than one contact, 34% (27 *100/79) with two, 15% (12 *100/79) with three and 5% of hot spots with more than three contacts in good agreement with observed values.

We then looked whether the hot spots had unusual amino acid features according to their number of contacts. The frequency of a hot spot in multiple contacts is divided by its frequency in single contact to measure the amino acid propensity to have multiple contacts. This propensity is plotted against the respective number of atoms of the side chain. No correlation is found for the BB hot spots (not shown, R = 0.41) and only branched residues V, I and L have a higher tendency of making two interactions suggesting that they are enriched in intermolecular β-strands involving parallel β-strands. On the other hand, there is a good linear correlation for the SC hot spots (Fig. 5B, R = 0.8). Thus, the propensity of the SC hot spot to make contacts is proportional to the number of its side chain atoms. Lastly, the probability of having hot spots with more than three contacts (*k*>3) is plotted against the number of atoms and compared to the probability of having a hot spot with one contact only (Fig. 5C). The probability of having hot spots with more than three contacts increases with the number of atoms whereas the probability of single hot spots distributes around a probability equal to 0.05. This probability (1/20) implies identical chance for all amino acids to have a single contact indicating no amino acid specificity for such contact number. On the other hand, only residues with more than 14 atoms (F, Y, R and W) have a probability above 0.05 to make more than three contacts, with the exception of the residue K.

## Discussion

The analysis of the individual hot spot properties confirms a scattered charge distribution on the β-strands, high β-sheet propensity residues enriched centrally and more particularly branched side chain residues (V and L). This indicates that linear information, namely the information read on the sequence of the β-strands, codes essentially for solubility and regulation of the secondary structures.

Discriminating SC and BB interactions is again relevant at the level of the pairs as the SC and BB pair preferences diverge significantly. The ratio of SC and BB hot spots and the ratio of SC and BB pairs are on average around 2, indicating that the SC preferences are likely to have more influence over the intermolecular β-strands. One novel observation is that the pair matching is not only based on the chemistry of the amino acids but also on their geometry as seen in the preferences for long or charged residues in the SC pairs and for small or hydrophobic residues in the BB pairs. There is even enrichment in pairs combining amino acid properties such as pairs between long and charged residues or pairs between long and polar residues. In both SC and BB pairs, the branched residues V, I and L are preponderant contacts. A chemical-centric view for the pair matching is obviously ill-appropriate and in fact the pairing calls upon the versatile properties of amino acids. It might be interesting to explore the role of geometrical parameters on the formation of intermolecular β-strands, experimentally and theoretically. For instance, one theoretical approach would be to use Minimum Steiner trees which offer a purely geometrical description of the amino acids, to determine whether the pair matching yields a minimum energy conformation of the interface [49]. Contacts between identical residues represent only around 10% of the total preferred contacts indicating a minor role in the matching process. This differs from previous report on dimeric intermolecular β-strands and from the prediction by a PIRA model [25], [31]. The data show that the 2D information, namely the amino acid pairing is not random and is important for the intermolecular β-strands, not surprisingly since β-strands are not viable without making interactions with another structural element.

Now the SC and BB networks do not differ only by their amino acid pairing but also by distinct network features. The BB networks have nodes with single or two contacts probably reflecting the hydrogen bond networks of anti-parallel (single contact) and parallel β-sheets (two contacts), respectively. The BB networks would essentially code for secondary structure interactions. The SC networks follow an exponential decay degree distribution and have nodes with one, two or three contacts but rarely with more than three. Thus the intermolecular β-strands result from the juxtaposition of two networks and the information for making the interface is encoded via a double layer of interactions. One layer is composed of the BB atoms and provides promiscuous interactions, namely low specificity in terms of amino acid composition and interaction motifs. The second layer is composed of the SC atoms which on the contrary provide selective interactions, high specificity in terms of amino acid composition and interaction motifs. Such type of double layer of interactions has been depicted for the interfaces between colicins and their immunity binding proteins as a way to evolve binding affinity [50]. There is also a precedent describing monomeric proteins and intramolecular amino acid interaction networks [51]. One network, based on short range interactions between Cα, had a bell curve degree distribution (random network feature) whilst the other based on long range interactions (side chain atoms) had an exponential decay degree distribution (single-scale network feature).

The exponential decay degree distribution likely fits a network optimized to reduce the number of links, relevantly because it costs to make a chemical link. Moreover, the data shows that above three contacts there is a strong stringency on the choice of the amino acids, suggesting that a node with too many links, a hub, would seriously decrease the sequence plasticity to successfully realize an interface. Intermolecular β-strands are very plastic in term of sequence requirement and seem therefore built to avoid hubs. Hubs are communication devices but also the Achilles' heel of the network: a modification of a hub spreads changes within the whole network because the hubs are connected to many nodes [52]. The propagation of changes upon node modification is called network rewiring [53]. The intermolecular β-strand networks which lack hubs are likely little inclined to rewiring because of their low interconnectedness. Counterintuitively, the robustness of intermolecular β-strands would appear based on a weak occurrence of links maintaining high sequence plasticity, cutting costs in term of links and reducing their vulnerability to changes (mutation).

**Figure 6:**
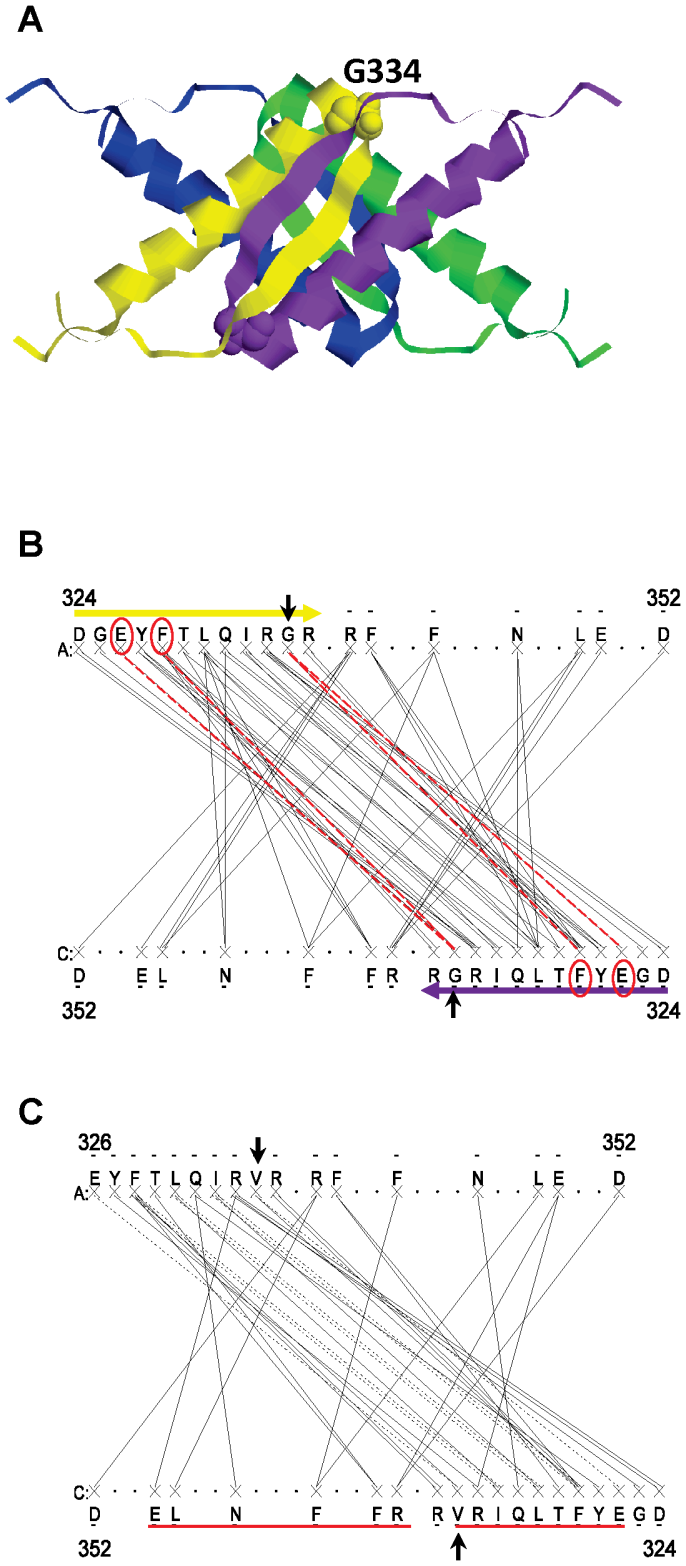
The p53 intermolecular β-strand network. A. Atomic structure of the p53 tetramerization domain (PDB 1SAK). The picture is generated with Rasmol, the four chains are shown in different colored ribbons. The G334 residue is indicated in spacefill. B. Gemini graph of the WT p53 tetramerization domain. The intermolecular β-strands composed of the residues 324 to 334, are highlighted by the yellow and purple arrows. The vertical arrows point to the residue 334. The links and hot spot contacts of G334 are shown by dotted red lines and red circles, respectively. C. Gemini graph of the G334V mutant. The hot spots whose links are affected by the mutation are underlined in red. The changes are not limited to residues in direct contact with G334 or to residues of the intermolecular β-strands.

It is tempting to speculate that a higher number of links is one of the necessary conditions to have a transition from “functional” to “aberrant” intermolecular β-strands. It is possible that “healthy” protein oligomers which become pathological fibers have interfaces with more links per nodes and networks more sensitive to rewiring than those which do not form fibers. To examine such possibility, the tumor suppressor p53 tetramer (PDB 1SAK, fig. 6A), a known case of healthy oligomer undergoing a transition to a fiber is considered. First, the Gemini graph of the WT p53 is generated (Fig. 6B). The greater occurrence of multiple contact residues is striking in the WT p53 network, supporting the hypothesis. The p53 hot spots have on average <*k*> = 3 contacts, twice the <*k*> value of the intermolecular β-strand networks. The p53 network has 33% hot spots with more than three contacts which is 6 times more than the prototype network. On the other hand, it has 25% of single contact hot spots twice less than the prototype network. Consequently the interconnectedness is larger in the p53 network than in the prototype network.

To look at the sensitivity of the p53 network to single point mutation, the G334V mutant, a familial mutation that leads to the dissociation of the p53 tetramer, misfunctions of the protein and cancer development, is considered [54]. The Gemini graph of G334V is generated and network rewiring is investigated (Fig. 6C). The mutation has a strong global effect on the network as all the residues of the p53 intermolecular β-strands from 324 to 334 have their links modified by the mutation even when they are not directly linked to the residue 334. The modifications are either: (i) vanishing of the links (e.g. D324, G325), (ii) changes of the type of links such as side chain to backbone (e.g. I332, L330), (iii) decrease of the number of contacts (e.g. Q331, T329) or else (iv) changes of contacts (R333). The changes in the network are not limited to residues of the intermolecular β-strands but extend to interactions between residues that belong to α-helices. This definitely shows that there is significant network rewiring in p53 due to a single node modification, the mutation of the residue G334, again supporting the hypothesis. Mutation of other p53 residues such as T329A or Q331A also leads to similar network rewiring (not shown) which therefore cannot explain the capacity of the mutant G334V to form a fiber, because the T329A and Q331A mutants do not make to fiber [54]. The extent of the changes in the network might be such that the intermolecular β-strand interactions are destabilized promoting chain dissociation, the first step to fiber formation.

### 

#### Conclusion

The key results are: (i) little information is accessible from individual amino acids (i.e. in sequences) and it is the pairs of amino acids that need to be investigated, (ii) the geometry of the amino acid side chains, so far neglected, is a key parameter to understand pair matching and finally (iii) intermolecular β-strands need to be further explored in terms of networks. The intermolecular β-strand networks are rather disconnected networks with no hubs but nodes with few links instead. Such a layout has several advantages as already discussed but probably the most relevant one is the secluding characteristic of the network which may well serve to limit the spread of changes, namely the rewiring, and protect the interface from dissociation upon mutation.

## Methods

### Definitions

#### Graph

graph, or a network, is a set of many components that interact with each other through pairwise interactions. At a highly abstract level, the components can be reduced to a series of nodes that are connected to each other by links, with each link representing the interactions between two components. The nodes and links together form a network, or, in more formal mathematical language, a graph [55]. The terms nodes and links used in graph theory are amino acids/hot spots and contacts/interactions, respectively, in the present context. The number of links of a node is the degree *k* of the node. In the networks of hot spots in interaction, the residues are connected through different motifs. Two residues connected by only one link make a single pair while two residues connected by more than one link make a multiple pair. Hot spots involved in single pair are single contact hot spots. Hot spots with more than one individual contact are called multiple contact hot spots.

#### Global propensity (GP)

The global propensity of an amino acid is the ratio of its frequency in a defined environment by its frequency in a database. Here the global propensity measures the frequency of every amino acid in intermolecular β-strands divided by its frequency in the whole chain.

Local preferences: the local amino acid preferences measure the preferred position of every amino acid on the β-strands. It is calculated as the difference of the frequency of a hot spot at the β-strand extremities (outer position) and its frequency when centrally located (any other position) on the strand.

Chemistry of the side chain of amino acid: charged amino acids are D, E, H, R and K; polar amino acids are N, Q, S, T and Y; hydrophobic residues are A, C, F, G, I, L, M, P, V and W.

Length of the side chain of amino acid: long side chain residues are K, W, R and K; medium side chain residues are D, N, L, I, H, E, Q, M and F and short side chain amino acids are G, A, P, C, S, T and V.

## Methods

### Construction of a non-redundant dataset

The Protein Data Bank (PDB) was first screened at the Research Collaboratory for Structural Bioinformatics (RCSB) for protein oligomers of stoichiometry above 2 and lower or equal to 12 [56]. Above dodecamers the number of cases becomes small for statistical analysis. Dimers are excluded from the dataset because of their diversity of orientation contacts implying broad diversity in recognition contact modes [57]. Viral and membrane proteins have been removed because they are likely to follow a different mechanism of interface formation than soluble oligomers. The coordinates of biological assembly were taken to select for non-crystallographic oligomers. NMR and X-ray structures are taken into account. PDB entries containing only backbone (BB) atoms, or only a few side-chain (SC) atoms, are discarded by monitoring the ratio of available SC and BB atoms for each of the twenty amino acids. Proteins with sequences similar at 90% identity are removed. As a result, 6234 PDBs have been tentatively treated with Gemini to describe the whole interface. There is a small minority of cases where Gemini stops before yielding the interface. Mainly, this is due to the presence of a single subunit in the PDB file, while Gemini expects several. This happens even if biological assemblies were downloaded from the RCSB. At this point, the interface is available for a set of 5248 proteins. Receptor-ligand, enzyme-inhibitor, and antigen-antibody types of interactions involve different ranges of K_D_ than permanent oligomers and as such are expected to have different recognition modes [42]. Therefore they are discarded from the dataset by removing proteins having at least one very short chain (≤20 amino acids). Truncated proteins were also discarded from the dataset by selecting only cases having chains less than 20 amino acid different in length.

Using the secondary structure annotation provided in the PDB file, the cases with intermolecular β-strands were extracted according to the following set of rules (to be simultaneously satisfied): 1) at least 3 bonds must be between amino acids belonging to β-strands; 2) at least 2 interface amino acids of each subunit must be in a β-β bond; 3) at least 5 interface amino acids must be classified β. The first rule is actually redundant as it is implied by the second and the third. To simplify the treatment, in the case of hetero-oligomers with more than one intermolecular β-strand, only one, randomly chosen, has been considered. The final list has been screened against redundancies by mapping each PDB code into a UniProt identifier. This allows using the appropriate UniProt algorithms to find and remove redundant cases. After this final suppression, we are left with 755 proteins having 1048 regions of intermolecular β-strands.

### Hot spots in interaction

A pair of hot spots is made of a hot spot –*a*- interacting with a hot spot –*b*-. Some hot spots participate in more than one pair at the same time and it is necessary to avoid their multiple counting. A pair (A1, A2) is counted 1/n time with *n* the number of bonds of A1. Let's consider a hot spot G forming a pair with T and another pair with L. Each of the (G, T) and (G, L) pairs is counted a half so the occurrence of G is equal to one and not to two if the pairs (G, T) and (G, L) had been counted one each instead of a half. This counting procedure implies that the tables of occurrences must be read row-wise (Tables 5 and 6). Now, when the number of interactions (bonds) issued from a hot spot is counted instead of the pair occurrences such normalization is unnecessary.

### Statistical tools

χ^2^. *n_ab_* and *n_ba_* pair occurrences. The total observed pair occurrences *n_ab_* and *n_ba_* are calculated for each residue as the sum of the occurrences on a row and the sum of the occurrences on a column (Tables 5 and 6 for the BB and SC sub-networks, respectively). The significance of the differences of the occurrences *n_ab_* and *n_ba_* was assessed using a χ^2^ (equation 2) with one degree of freedom calculated as follows:
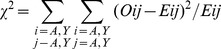
(2)


With O_ij_ the observed occurrences (line i and column j on the tables 5 and 6) and E_ij_ the expected occurrences calculated as the average value of the total observed pair occurrences *n_ab_* and *n_ba_*. The sums are for the *n_ab_* and the *n_ba_* occurrence values. For one degree of freedom, a χ^2^ value inferior to 3.84 is not significant (5% threshold significance).

Observed (*f_ab_*) and expected values (*f_a_*×*f_b_*). The significance of the differences of the observed (*f_ab_*) and expected pair frequencies (*f_a_*×*f_b_*) was also assessed using a χ^2^ with O_ij_ and E_ij_ the observed and expected pair frequencies, respectively. This time it is calculated over a matrix where low occurrences (below 5) are summed and a p-value is calculated.

#### Binomial law

This law calculates the probability of making a link *P(k)* over a large number of test *n* with *p* the probability to make a link and (1-*p*) the probability to make no link (equation 3). Thus the probability of any SC hot spot to make *k* links (i.e. *k* number of contacts) is calculated as the product of the probability for any node to make *k* links by its probability to make no link over *n* trials. When the calculated values are close to the observed values, the binomial law is a good model for estimating the number of links of the hot spots.
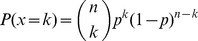
(3)


### Virtual mutation

Fold X is used to generate the virtual mutation G334V in the PDB of the p53 tetramerization domain was designed following instruction in [58], [59].

### Availability of supporting data

The list of the 755 PDB cases and their respective intermolecular β-strands are available on request.
